# Wnt/β-catenin-driven EMT regulation in human cancers

**DOI:** 10.1007/s00018-023-05099-7

**Published:** 2024-02-09

**Authors:** Wenhua Xue, Lin Yang, Chengxin Chen, Milad Ashrafizadeh, Yu Tian, Ranran Sun

**Affiliations:** 1 Department of Pharmacy, The First Affiliated Hospital of Zhengzhou University, Zhengzhou, 450052 Henan People’s Republic of China; 2 Department of Hepatobiliary Surgery, Xianyang Central Hospital, Xianyang, 712000 Shaanxi China; 3 Shanghai Institute of Cardiovascular Diseases, Zhongshan Hospital, Fudan University, Shanghai, 200032 China; 4 School of Public Health, Benedictine University, Lisle, USA; 5 Precision Medicine Center, The First Affiliated Hospital of Zhengzhou University, Zhengzhou, Henan China

**Keywords:** EMT, Cancer metastasis, Wnt/β-catenin, Chemoresistance, Phytochemicals

## Abstract

Metastasis accounts for 90% of cancer-related deaths among the patients. The transformation of epithelial cells into mesenchymal cells with molecular alterations can occur during epithelial–mesenchymal transition (EMT). The EMT mechanism accelerates the cancer metastasis and drug resistance ability in human cancers. Among the different regulators of EMT, Wnt/β-catenin axis has been emerged as a versatile modulator. Wnt is in active form in physiological condition due to the function of GSK-3β that destructs β-catenin, while ligand–receptor interaction impairs GSK-3β function to increase β-catenin stability and promote its nuclear transfer. Regarding the oncogenic function of Wnt/β-catenin, its upregulation occurs in human cancers and it can accelerate EMT-mediated metastasis and drug resistance. The stimulation of Wnt by binding Wnt ligands into Frizzled receptors can enhance β-catenin accumulation in cytoplasm that stimulates EMT and related genes upon nuclear translocation. Wnt/β-catenin/EMT axis has been implicated in augmenting metastasis of both solid and hematological tumors. The Wnt/EMT-mediated cancer metastasis promotes the malignant behavior of tumor cells, causing therapy resistance. The Wnt/β-catenin/EMT axis can be modulated by upstream mediators in which non-coding RNAs are main regulators. Moreover, pharmacological intervention, mainly using phytochemicals, suppresses Wnt/EMT axis in metastasis suppression.

**Graphical abstract:**

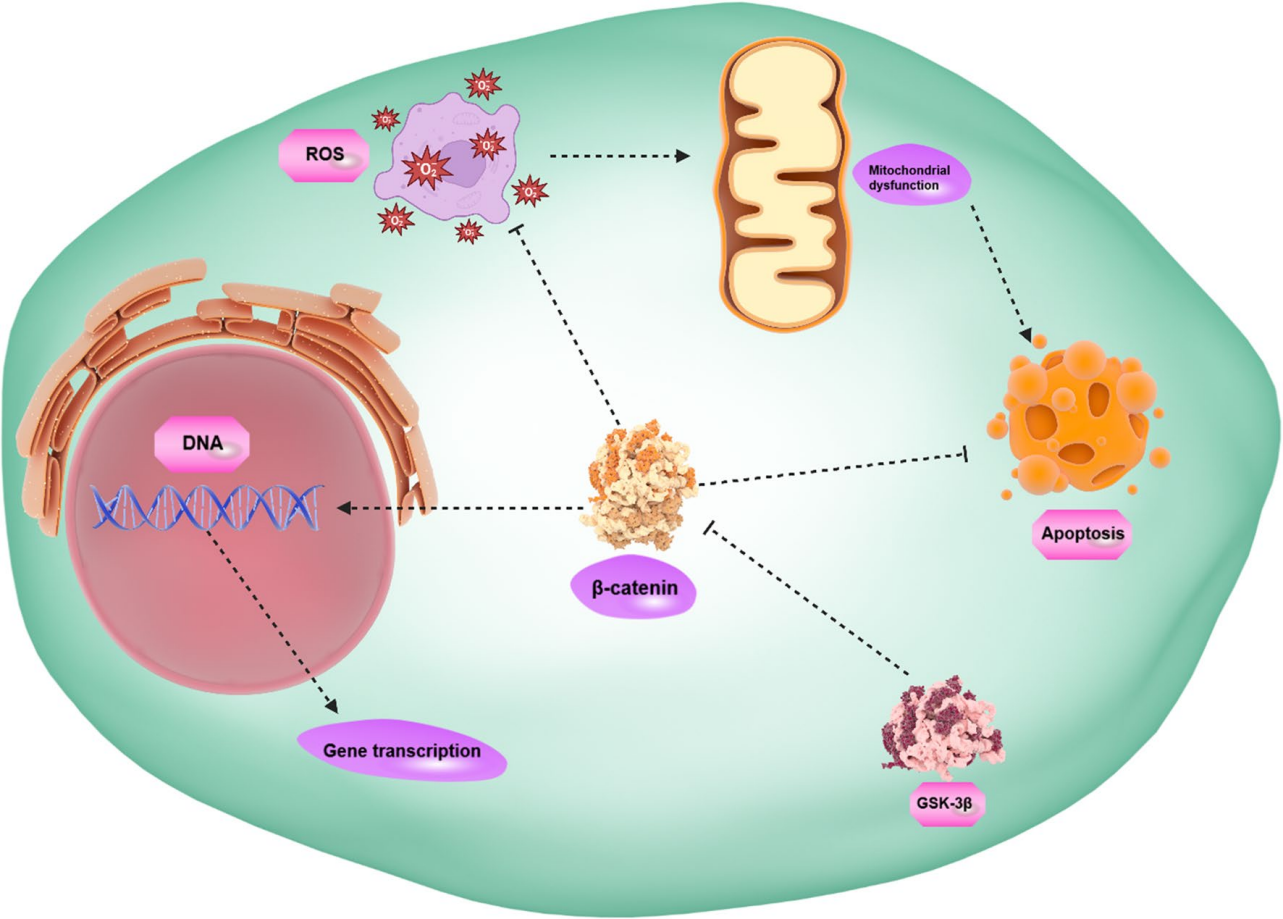

## Introduction

Wnt/β-catenin is an evolutionary conserved pathway essential for embryonic development and regulation of adult stem cell, homeostasis and tissue regeneration [1–5]. Pre-clinical and clinical evidences have confirmed the vital role of Wnt in the initiation and development of human diseases, particularly cancer [6]. Up to date, 19 glycoproteins have been identified in Wnt ligand family to function in a paracrine or autocrine manner and demonstrate various spatiotemporal expression. Upon their synthesis, Wnt proteins are transferred to lumen of endoplasmic reticulum (ER) to undergo palmitoylation by PORCN in ER. Then, Wnt ligand interacts with Wntless (WLS) to accelerate release of Wnt proteins [4]. Passive diffusion or secretion in membrane-enclosed vesicles or traveling by cytonemes are responsible for cell–cell transfer of Wnt ligands [5, 7]. Wnt has two pathways including canonical and non-canonical. The canonical pathway occurs in self-renewing and undifferentiated state triggered by addition of Wnt3a to cell culture [8–11], while in the non-canonical pathway, Wnt5 ligand is capable of promoting differentiation and migration, curtailing proliferation [12, 13]. The interaction of Wnt receptor with ligands is responsible for its stimulation accounting for transmission of cell signaling information from extracellular environment to intracellular compartments and downstream targets [14]. Wnt is tightly controlled in cells and is an important participant in physiological processes including organism axis differentiation, tissue formation, brain formation and stem cell maintenance [15, 16]. Accumulating data has shown association of Wnt dysregulation with the development of pathological events [17, 18]. On the cell surface, there are receptors including LRP5/6 and FZD that can interact with Wnt ligands to induce Dishevelled protein, a member of complex with other proteins including GSK-3β, Axin2 and APC. Then, accumulated β-catenin transfers to nucleus to stimulate TCF and LEF family members in further modulation of gene targets [19]. However, there is non-canonical and β-catenin-independent pathway including Wnt/Ca^2+^ signaling, mTOR signaling, Ras homolog gene family, RhoA/ROCK and JNK signaling cascades [20]. The Wnt inhibitory factor (WIF) or SFRPs can bind to Wnt ligands or interaction of DKK1 or SOST with LRP5/6 receptor can occur to suppress Wnt signaling [21].

**Fig. 1:**
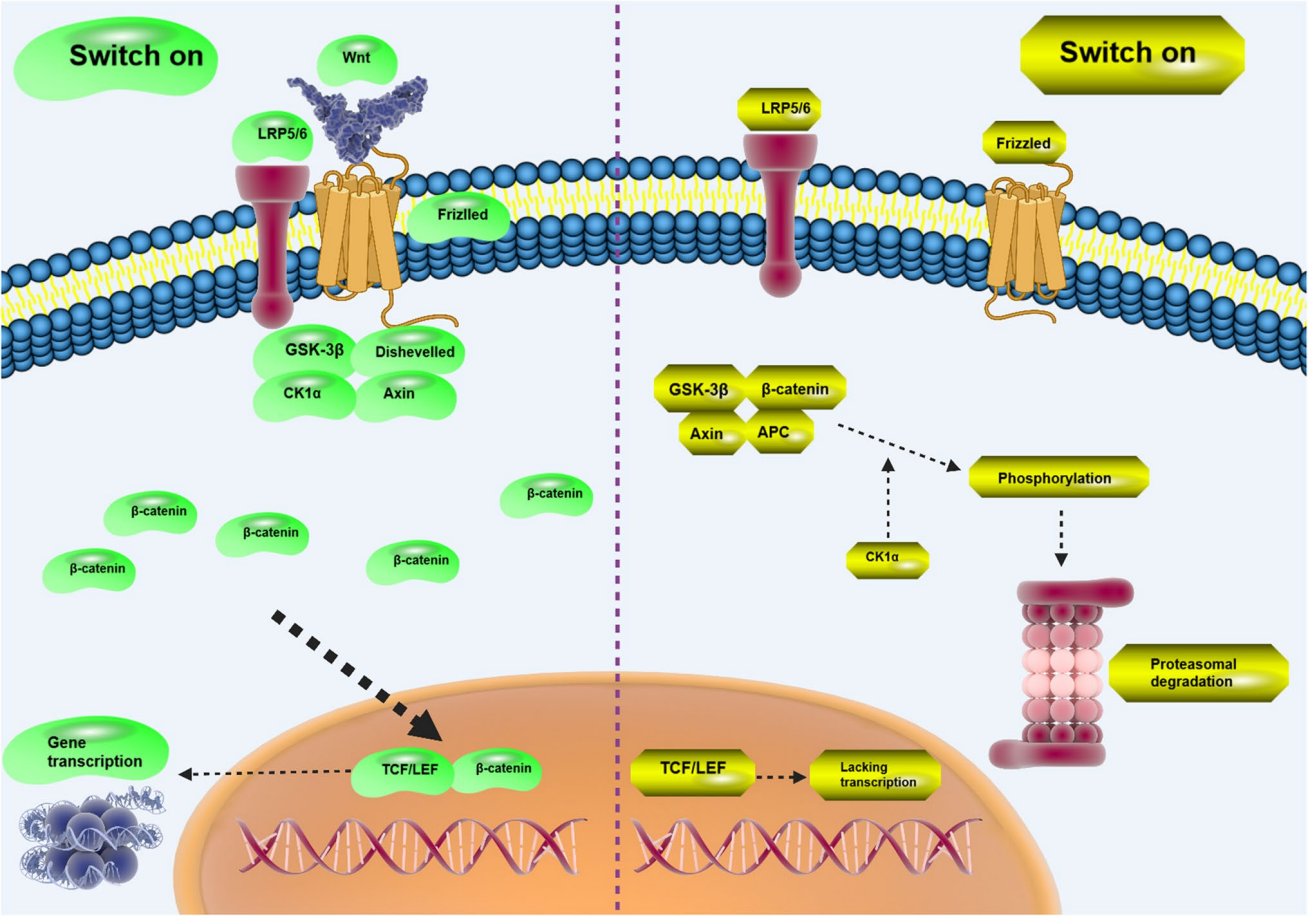
The presence of Wnt ligand mediates the function of Frizzled and LRP5/6 receptors to suppress GSK-3β, Dishevelled, CK1α and Axin. Then, it enhances the intracellular accumulation of β-catenin to transfer into nucleus and enhance gene transcription. However, lack of Wnt ligand mediates the phosphorylation of β-catenin to enhance its proteasomal degradation

Figure 1 represents Wnt and related molecular interactions. Wnt has been associated with the development of various human cancers. Wnt/β-catenin overexpression occurs in thyroid cancer due to KDM1A upregulation to increase cancer malignancy and promote stemness [22]. Upregulation of Wnt can lead to resistance of tumor cells to cell death. For instance, overexpression of Wnt/β-catenin promotes GPX4 expression to induce ferroptosis resistance in gastric cancer cells. Silencing GPX4 enhances sensitivity to ferroptosis [23]. LINC01606 also adopts a similar manner in the regulation of cell death in colon cancer. By promoting SCD1 expression, LINC01606 induces Wnt to prevent ferroptosis in tumor cells [24]. Interestingly, metabolism of tumor cells can be controlled by Wnt. NME7 as a protein kinase is able to induce Wnt/β-catenin to enhance one-carbon metabolism in hepatocellular carcinoma [25]. A synergy between Wnt/β-catenin and PI3K/Akt is able to enhance HIF-1α expression in glycolysis induction and stimulation of 5-flourouracil resistance in colorectal tumor [26]. The interactions of upstream mediators can determine progression of tumor cells via Wnt/β-catenin control. Circ-EIF6 is capable of encoding EIF6-224aa in enhancing tumor progression. The downstream signaling is unique and by stabilizing MYH9, circ-EIF6 participates in the induction of Wnt/β-catenin and elevation of breast cancer progression [27]. Both carcinogenesis and drug resistance can be modulated by Wnt/β-catenin in cancers. N^7^-methylguanosine tRNA modification stimulates Wnt/β-catenin in inducing drug resistance and increasing progression in nasopharyngeal cancer [28]. Upon deficiency of TET, Wnt is reprogrammed to impair its promoter demethylation and enhance lung cancer malignancy [29]. Based on these findings, interaction of Wnt with other molecular pathways determines cancer progression and its targeting is of importance in cancer therapy [30–33]. Table 1 summarizes the recent findings of Wnt dysregulation in human cancers. In the current review, we will focus on revealing function of Wnt in regulating tumor metastasis by affecting a well-known mechanism, epithelial–mesenchymal transition (EMT).

**Table 1:** Dysregulation of Wnt axis in human cancers

Human cancer	Molecular profile	Highlight	Refs.
Laryngeal cancer	MAGP1/Wnt	MAGP1 stimulates Wnt/β-catenin to enhance MMP-7 levels Stimulation of angiogenesis	[34]
Gastric cancer	Wnt/GPX4	β-catenin/TCF4 transcription binds to the promoter of GPX4 and increases its expression to mediate ferroptosis resistance	[23]
Breast cancer	WNT	The p53 deficiency stimulates WNT-mediated systemic inflammation, aggravating tumor metastasis	[35]
-	WNT	The high immunogenic tumor cells use WNT pathway upregulation for mediating immune evasion	[36]
Head and neck cancer	CMTM6/ENO-1/Akt/GSK-3β	CMTM6 stimulates cisplatin resistance through stabilization of ENO-1 and subsequent upregulation of Wnt	[37]
Colon cancer	Drp1/Wnt	The upregulation of Drp1 enhances the fatty acid-mediated metabolic reprogramming to accelerate Wnt activation	[38]
Gastric cancer	TNFRSF11B/Wnt	TNFRSF11B enhances Wnt/β-catenin expression to accelerate tumorigenesis	[39]
Colorectal cancer	Wnt	The application of MEK inhibitors can stimulate Wnt axis and enhance stem cell plasticity	[40]
Breast cancer	RCC2/Wnt	RCC2 upregulates Wnt to induce EMT-mediated cancer metastasis	[41]
Intestinal cancer	Wnt	PROTAC as a peptide enhances β-catenin degradation to impair Wnt-mediated intestinal cancer progression	[42]
Bladder cancer	RSPO3/Wnt	RSPO3 upregulates Wnt and Hedgehog to increase tumorigenesis	[43]

## EMT mechanism: general aspects and carcinogenic function

Complexity in metastasis process has been intriguing to researchers and the mechanism of metastasis can be observed in various human cancers to disseminate in other tissues and increase their population. Moreover, metastasis has reverse relationship with prognosis and overall survival of patients. The metastasis program can be simply defined as detachment of some tumor populations from their colony to enter into bloodstream and then, exit from circulation to a new tissue and establishing a new colony. In contrast to simple definition of metastasis, molecular interactions participating in metastasis are rather complicated. Despite the recent advances in metastasis research, there are still many unidentified aspects of cancer metastasis at molecular and cellular levels. In order to better understand metastasis, key molecular processes have come into attention, among which epithelial–mesenchymal transition (EMT) being an attracting mechanism. Generally, loss of polarity in epithelial cells and obtaining mesenchymal-like features are known as EMT that there are different categories of EMT based on occurring in embryogenesis, wound healing/tissue fibrosis and tumor cells [44, 45]. The tight junctions, desomosomes, gap junctions and adheren junctions are responsible for attaching epithelial cells to basement membrane [46]. However, during maturation in the developmental process, these cells become more motile and lose their polarity [47]. A similar process can be observed in cancer cells where they are separated from basement membrane and enter into bloodstream, resulting in increased migration and metastasis [48]. E-cadherin down-regulation, and vimentin and N-cadherin upregulation can be observed during loss of polarity in epithelial cells. The EMT-inducing transcription factors (EMT-TFs) are present and demonstrate high expression in tumor cells such as TGF-β, ZEB proteins, Slug and Twist to increase cancer metastasis [49, 50].

In the recent years, the factors regulating EMT in cancer metastasis have been of interest with new insights and ideas being offered towards the EMT modulators. During hypoxia, expression level of Nrf2 increases through down-regulation of miR-27a. Nrf2 participates in EMT induction and elevating metastasis of lung tumor cells [51]. Loss of expression of iNOS/NO is involved in elevating invasion of colorectal tumor cells via EMT induction [52]. The interesting point is that EMT is not certain to a tumor type and its regulation by various factors can affect carcinogenesis and metastasis. PODNL1 exhibits high levels of expression in bladder cancer. By reducing E-cadherin levels, PODNL1 promotes EMT in tumor cells [53]. On the other hand, there are factors that can suppress EMT in cancers. For instance, FOXA2 has been in favor of decreasing cancer metastasis and it suppresses EMT [54]. Since molecular pathways regulating EMT mechanism have been somewhat well understood [55], studies have focused on EMT targeting in cancer therapy [52]. Decrease in N-cadherin and vimentin levels, and increase in E-cadherin levels can be observed after administration of β-elemene in inhibiting EMT in colorectal tumor [56]. As mentioned, hypoxia is involved in EMT induction and facilitated cancer invasion which can be overcome by β-patchoulene [57]. Moreover, there is a close association between EMT induction and drug resistance in human cancers [58]. Noteworthy, inhibition of Wnt/β-catenin by fucoxanthin can suppress EMT in lung cancer, suggesting the role of Wnt as a regulator of EMT [59] (will be discussed in details in next sections). Table 2 summarizes the role of EMT in human cancer invasion.

**Table 2:** The dysregulation of EMT in human cancers

Human cancer	Molecular profile	Highlight	Refs.
Ovarian cancer	Ets1/Drp1/EMT	Ets1 increases Drp1 levels to induce EMT for enhancing metastasis	[60]
Endometrial cancer	Netrin-1	The inhibition of Netrin-1 impairs the proliferation and EMT	[61]
Bladder cancer	CircPTK2/PABPC1/SETDB1	CircPTK2 increases stability of PABPC1 to upregulate SETDB1, causing EMT and accelerating cancer metastasis Stimulation of gemcitabine resistance	[62]
–	RHOJ/EMT	RHOJ controls the EMT-induced drug resistance through increasing response to replicative stress and stimulation of DNA damage response	[63]
Colorectal cancer	DDX21/MCM5/EMT	DDX21 increases MCM5 levels to induce EMT-mediated cancer invasion	[64]
Pancreatic cancer	PYGL	PYGL stimulates the reprogramming in glucose metabolism to induce EMT and accelerate invasion	[65]
Triple-negative breast cancer	TRAIP/EMT	TRAIP knock-down impairs the growth and metastasis of tumor cells	[66]
Pancreatic cancer	STMN2/Wnt	STMN2 is able to stimulate EMT and increase cancer progression through Wnt upregulation	[67]
Colorectal cancer	–	Delivery of shikonin by nanoparticles can impair metastasis through EMT inhibition	[68]

## Wnt/β-catenin signaling as a regulator of EMT in human cancers

### Brain tumors

Glioblastoma (GBM) is a cancer of central nervous system [69] with an incidence rate of 7.2/100,000 [70]. GBM causes high mortality rate and the survival rate of patients appears to be less than 14 months [71]. Surgery, radiotherapy and chemotherapy are considered as current treatments for GBM [72] and in addition to poor efficacy of current therapies, it is quite troublesome to eliminate GBM by surgical resection [73]. The aggressiveness of GBM cells depends on EMT induction and invasion, and STAT1 upregulation is essential in this case. STAT1 is capable of evoking Wnt/β-catenin axis in EMT induction and elevating invasion and metastasis of GBM cells [74]. Since stimulation of Wnt enhances GBM metastasis, its inhibition will have negative impact on the aggression of tumor cells. This has been evaluated in a recent experiment that LINC-PINT impairs Wnt/β-catenin axis to suppress EMT in decreasing metastasis of GBM [75]. More importantly, when transcription of FZD7 occurs, it can interact with Wnt ligand to induce β-catenin translocation to nucleus. If its transcription is suppressed, there will be little receptor for ligand recognition. miR-504 reduces FZD7 expression to suppress β-catenin in suppressing EMT and stemness in GBM [76]. In order to disrupt progression of GBM cells, studies have focused on knock-down of factors responsible for EMT stimulation in cancers. For instance, when expression level of GOLM1 decreases and it is silenced, suppression of Wnt/β-catenin axis occurs that is vital for reducing metastasis of GBM cells [77]. Therefore, knock-down and upregulation of certain upstream factors regulating Wnt/EMT axis can help in highlighting the mechanisms involved in GBM aggression [78]. Interestingly, high expression of CTNND1 is vital for the induction of Wnt/β-catenin in GBM and subsequent EMT induction [79]. Therefore, studies have highlighted the function of Wnt/EMT axis in aggravating GBM progression. However, GBM is not the only brain tumor and glioma is another one that researchers have also focused on Wnt/EMT function in its progression. Upregulation of LGR5 results in unfavorable prognosis in glioma. By inducing Wnt/β-catenin axis, LGR5 mediates EMT and promotes cancer invasion and metastasis [80]. One of the important targets in the treatment of brain tumors is CBX7 that its expression level is epigenetically reduced in tumor cells. Moreover, it is able to suppress cancer metastasis via inhibiting YAP/TAZ axis [81]. Furthermore, CBX7 can be considered as a prognostic factor in cancers. By reducing CCNE1 expression, CBX7 stimulates cell cycle arrest in glioma [82]. Similar to the approach that was followed in GBM therapy, for instance, silencing TRIM47 impairs Wnt pathway that is in favor of reducing vimentin and N-cadherin levels [83]. Hence, increasing evidence demonstrates that upregulation of Wnt can increase glioma progression via EMT induction [84, 85].

### Gastrointestinal tumors

The aim of current section is to evaluate role of Wnt/EMT axis in metastasis of gastrointestinal tumors. Gastric cancer is the third most common tumor around the world and the most common malignant of gastrointestinal tract in China [86–88]. However, metastasis results in recurrence in gastric cancer and that is why 5-year survival rate of patients is poor [89]. Samples obtained from gastric cancer patients revealed that ADMA serves as a potential factor for increased metastasis of tumor cells. By inducing Wnt/β-catenin, ADMA mediates EMT-induced metastasis [90]. The intriguing point is that factors and molecular pathways can regulate expression level of Wnt and nuclear translocation of β-catenin. However, a recent experiment has shown that Zic1 is a suppressor of β-catenin/TCF4 complex and does not affect the nuclear translocation of β-catenin. Then, it reduces expression levels of c-Myc and cyclin D1 as targets of Wnt and suppresses EMT in gastric tumor cells [91]. TIPE1 is a member of TNFAIP8 family and despite similarities in structure and sequence of TIPE family, their biological functions are distinct. TIPE3 and TNFAIP8 participate in the process of tumorigenesis [92, 93]; TIPE2 is a negative modulator of immune system and it is involved in inflammatory disease regulation [94, 95]; TIPE1 is a tumor suppressor-mediating apoptosis in cancer cells [96]. In gastric tumor, TIPE1 is an inhibitor of metastasis and for this purpose, it impairs Wnt/β-catenin to reduce levels of MMP-2 and MMP-9 in EMT inhibition and reducing malignancy [97]. The confirmation for role of Wnt in gastric cancer metastasis is that function of TFEB in enhancing invasion of gastric cancer and EMT induction is essential based on stimulation of Wnt [98]. Therefore, Wnt/EMT axis is an important pathway involved in enhanced invasion and metastasis of gastric tumor cells [99].

Colorectal cancer (CRC) is another important malignancy of gastrointestinal tract. Its morbidity and mortality ranked the 3rd and 2rd, respectively, among cancers, and is considered as a main threat to human health [100]. The incidence rate of CRC in young patients has demonstrated an increase [101] and survival rate of patients at stage I and II of CRC is 91% and 82%, for stage IV is 12% [102]. Similar to gastric tumor, one of the main problems of CRC is metastasis and therefore, studies have focused on understanding the role of Wnt/EMT axis in its progression and invasion. NCAPG may increase metastasis of CRC cells and when its expression enhances, it induces Wnt/β-catenin to mediate EMT in enhancing tumor invasion [103]. When Wnt undergoes activation in CRC, it can affect EMT-TFs including ZEB1. RHBDD1 upregulation in CRC results in phosphorylation of β-catenin to induce Wnt pathway for increasing ZEB1 expression and mediating EMT [104]. Moreover, Snail expression increases by Wnt in CRC cells and CEMIP down-regulation results in Wnt/β-catenin inhibition to reduce Snail expression in EMT inhibition [105].

Hepatocellular carcinoma (HCC) is another malignancy with an escalating incidence rate. It is one of the leading causes of death in Chinese population [106, 107]. Hepatitis B virus (HBV) is a leading cause of HCC around the world and up to 50–80% of cases are due to this infection [108]. Furthermore, by increase in number of NAFLD, incidence rate of HCC also increases [109]. High expression of Wnt in HCC can increase tumor progression, and aquaporin 9 impairs Wnt/β-catenin to suppress invasion and EMT in tumor cells [110]. A recent study revealed that increased expression of Wnt can be mediated by borealin (which induces β-catenin in EMT induction and promotes HCC invasion and metastasis) [111]. OCT4 is another factor related to stemness, but by activating LEF1/β-catenin axis, it mediates EMT in a Wnt-dependent manner [112].

### Urological tumors

Prostate cancer (PCa) is the sixth most common tumor in men and causes high mortality [113]. The major reason of PCa-related death is metastasis and inefficacy of current therapeutics [114]. FOXO3a is an inhibitor of metastasis in PCa and for this purpose, it suppresses EMT. The FOXO3a increases transcription of miR-34b/c in the nucleus and then, mature miR-34b/c in cytoplasm suppresses β-catenin to reduce ZEB/EMT. Moreover, FOXO3a can directly inhibit β-catenin. Therefore, FOXO3a may directly and indirectly suppress β-catenin/ZEB/EMT in reducing PCa progression [115]. GPX2, another factor mediating recurrence in PCa, has important function to promote metastasis of tumor cells mediated via β-catenin induction and subsequent increased cancer metastasis through EMT stimulation [116]. When silencing Wnt/β-catenin occurs, EMT-mediated metastasis in PCa is suppressed [117]. Interestingly, both TGF-β1 and Wnt can induce EMT in enhancing PCa metastasis. CD82/KAI1 functions as tumor suppressor that by inhibiting Wnt and TGF-β1, impairs metastasis in PCa cells and EMT mechanism [118]. Seventy percent of BC cases are non-muscle-invasive and metastasis is an increasing challenge in this case. Upregulation of Wnt results in EMT in BC, and EFEMP2 is capable of suppressing Wnt/β-catenin in reducing metastasis via EMT inhibition [119]. Another malignant urological tumor is renal cancer, although only one study has evaluated function of Wnt in the modulation of metastasis. In this case, Wnt increases levels of ARL4C to enhance cyclin D1 and c-Myc levels, leading to EMT induction and enactment in progression of tumor cells [120].

### Hematological cancers

Leukemia, lymphoma and myeloma are three important hematological tumors, but one of the drawbacks of current studies is ignorance towards understanding function of Wnt/EMT axis and only a few experiments have investigated that are included here. Since Wnt is an important regulator of tumorigenesis in these cancers, future studies should be engaged towards the role of Wnt/EMT axis in hematological tumor progression. PLAGL2 shows upregulation in lymphoma and by inducing Wnt/β-catenin axis, it mediates EMT to enhance invasion and metastasis of tumor cells [121]. Moreover, expression level of SOX12 decreases by miR-744-5p in myeloma and this results in Wnt/β-catenin inhibition in reversing EMT and decreasing invasion of tumor cells [122].

### Gynecological tumors

**Fig. 2:**
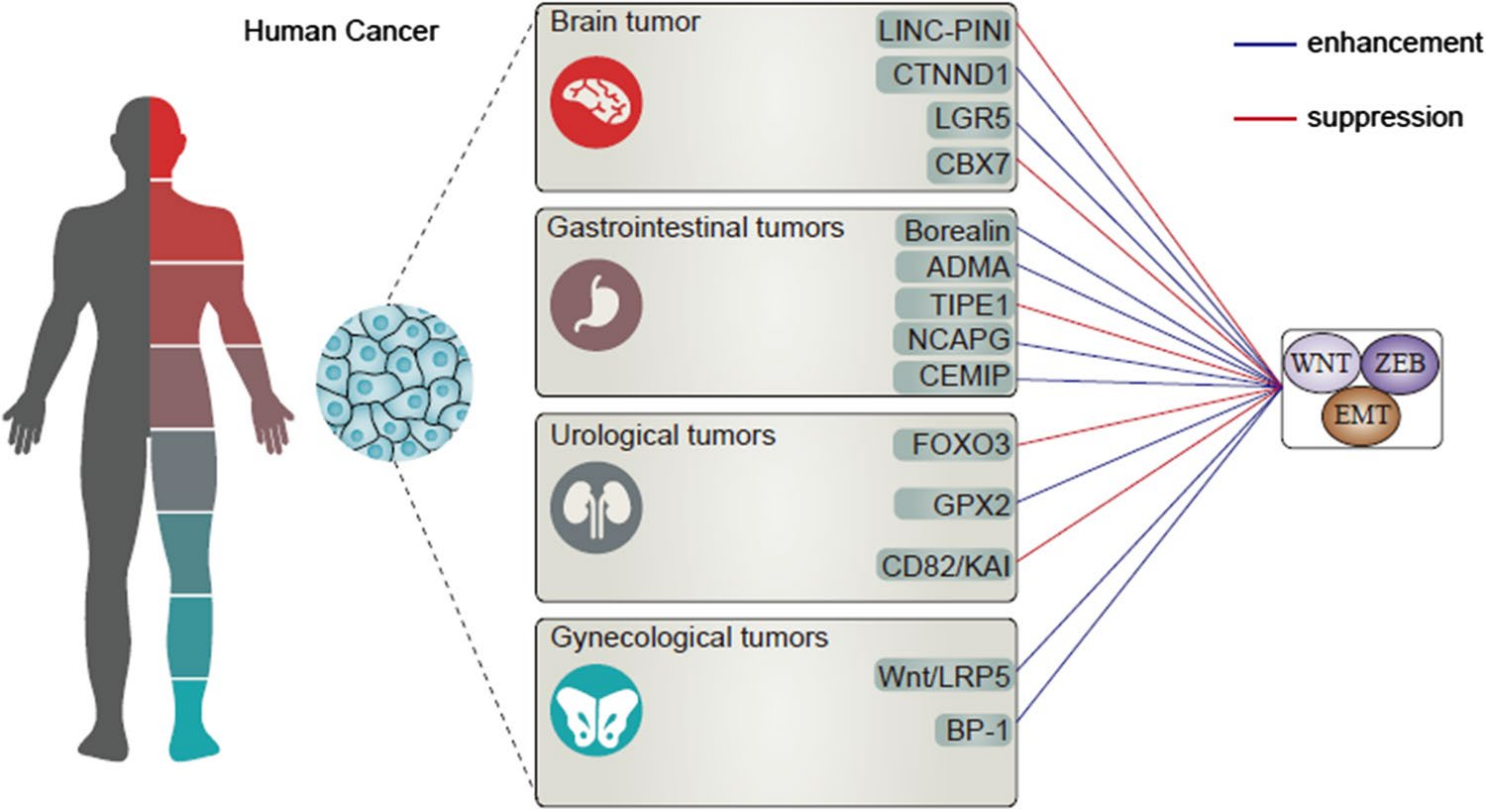
The aberrant levels of Wnt can cause progression of both solid and hematological tumors. Wnt interacts with ZEB and other EMT-TFs to mediate EMT. In each cancer, a number of related molecular interaction regulating Wnt/EMT axis have been identified

Ovarian cancer (OC) is another malignancy of gynecological tumors and the second leading cause of death [123]. The 5-year survival of patients in last 30 years has been less than 30% and up to 70% of females do not demonstrate symptoms in early stages; hence, peritoneal cavity metastasis is one of the major complications of OC [124]. The invasion of OC cells that can be accelerated by EMT is one of the reasons of OC malignancy. Inhibition of Wnt/LRP5 axis results in EMT inhibition and subsequent suppression of migration in vitro and in vivo [125]. One of the factors involved in cancer development is the exposure to chemicals and toxic agents such as benzophenone-1 (BP-1) with the ability of inducing Wnt/β-catenin and ERα expression to mediate EMT biomarkers [126]. Moreover, high levels of IL-8 as an inflammatory factor in OC can result in EMT stimulation via inducing Wnt/β-catenin [127]. Hence, targeting Wnt/EMT is promising in retarding OC progression. Another important gynecological malignancy is cervical cancer accounting for 6.5% of current cancer cases and 7.7% of female mortalities [128]. Inhibition of both Wnt and EMT can significantly diminish progression of cervical tumor cells [129]. However, the important part is related to role of Wnt/EMT as a novel axis in regulating cervical cancer progression. APMAP is deemed one of the key factors in cervical cancer metastasis, and stimulates EMT through induction of Wnt/β-catenin signaling [130]. Hence, both Wnt and EMT play a vital role in facilitated progression of cervical cancer cells [131]. The aim of “Wnt/β-catenin signaling as a regulator of EMT in human cancers” was to discuss the role of Wnt/EMT axis in various cancer types (as summarized in Table 3, Fig. 2).

**Table 3:** The role of Wnt/EMT axis in various human cancers

Pathway	Cancer type	Remark	Refs.
TET1/DKK1/EMT	Ovarian cancer	TET1 suppresses EMT and metastasis via increasing levels of DKK1 as Wnt inhibitor	[132]
CRIP1/Wnt/EMT	Ovarian cancer	CRIP1 stimulates Wnt/EMT axis in enhancing cancer invasion	[133]
CEBPA/Wnt/EMT	Ovarian cancer	CEBPA suppresses EMT via Wnt down-regulation in decreasing invasion	[134]
HOXB-AS3/Wnt/EMT	Ovarian cancer	HOXB-AS3 induces EMT via Wnt pathway	[76]
HOXC13-AS/β-catenin/EMT	Cervical cancer	HOXC13-AS induces EMT via mediating β-catenin	[135]
CRIP1/Wnt/EMT	Cervical cancer	CRIP1 induces EMT via triggering Wnt pathway	[136]
MAFE-A3/Wnt/EMT	Cervical cancer	MAGE-A3 stimulates Wnt in increasing invasion and mediating EMT	[137]
SFRP1/2/Wnt/EMT	Cervical cancer	SFRP1/2 suppress EMT via Wnt down-regulation	[138]
SMYD2/APC2/Wnt/EMT	Colorectal cancer	SMYD2 increases Wnt expression vi aAPC2 down-regulation to induce EMT	[139]
CD55/Smad4/β-catenin/EMT	Colorectal cancer	CD55/Smad4 suppresses β-catenin/EMT	[140]
HYD-PEP06/Wnt/EMT	Hepatocellular carcinoma	HYD-PEP06 inhibits Wnt/EMT axis in reducing tumor invasion	[141]
TNF-α/Wnt/EMT	Hepatocellular carcinoma	TNF-α secreted by M2 macrophages induces EMT via Wnt upregulation	[142]
Cx32/Wnt/EMT	Hepatocellular carcinoma	Low expression of Cx32 leads to Wnt/EMT	[143]
TRIM37/Wnt/EMT	Hepatocellular carcinoma	TRIM37 stimulates Wnt/EMT axis in promoting tumor progression	[144]

## Wnt/β-catenin signaling and cancer drug resistance via regulation of EMT

**Fig. 3:**
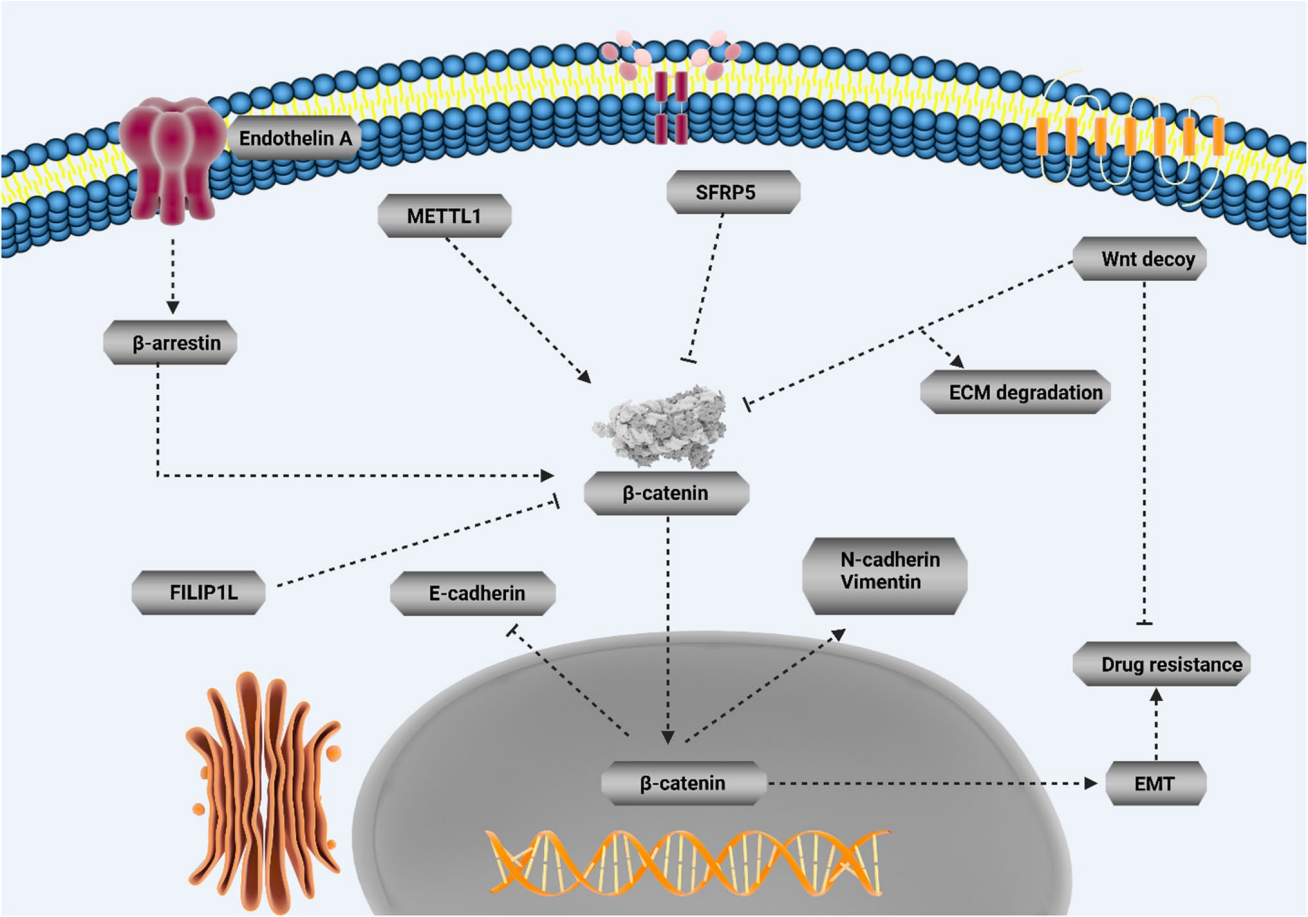
Wnt/EMT axis in cancer drug resistance. The nuclear transfer of β-catenin enhances N-cadherin and vimentin levels, while it downregulates E-cadherin to mediate EMT in cancer metastasis. Moreover, β-catenin can be upregulated by β-arrestin and METTL1 to mediate EMT and drug resistance

Drug resistance is an evolving field and imposes a major challenge for physicians around the world. Moreover, hallmarks of cancer can display association with chemoresistance. Since EMT increases metastasis as an important hallmark of tumors, the association of this pathway with drug resistance development has been evaluated in various studies. Moreover, overexpression of ZEB1 results in EMT-mediated drug resistance in ovarian cancer [145]. Therefore, if some factors suppress EMT, sensitivity of tumor cells to chemotherapy enhances. Upregulation of Par-4 decreases MDM-2 expression to suppress EMT-mediated drug resistance [146]. Moreover, miR-128-3p-mediated suppression of c-Met/EMT axis leads to increased temozolomide sensitivity in glioblastoma [147]. Therefore, EMT induction leads to chemoresistance, and this section focuses on the EMT regulation by Wnt in tumor cells and determines their sensitivity to chemotherapy. However, it should be noted that in all studies, the role of Wnt/EMT axis in the regulation of drug resistance has not been evaluated. For instance, it has been reported that endothelin A receptor/β-arrestin integrates with Wnt pathway to induce EMT and drug resistance in ovarian tumor [148]. Although previous study evaluated the role of Wnt and its correlation with other signaling pathways in EMT induction and chemoresistance, it is not certain that EMT leads to malignant progression and chemoresistance or not. However, since accumulating data has shown potential of EMT in increasing cancer aggressiveness, it can be indirectly concluded that EMT induction causes drug resistance. A similar approach has been followed in which METTL1 is able to induce Wnt/β-catenin in mediating chemoresistance and EMT nasopharyngeal cancer, but association of EMT and chemoresistance has not been evaluated [28]. However, there are studies showing that dysregulation of Wnt/EMT axis can lead to chemoresistance in human cancers. In pancreatic cancer, EMT and extracellular matrix (ECM) cooperate in the development of resistance to chemotherapy. Using an oncolytic adenovirus for expressing both Wnt decoy and decorin leads to ECM degradation and EMT suppression in suppressing gemcitabine resistance in tumor cells [149]. This experiment clearly demonstrates that Wnt inhibition can suppress EMT-induced chemoresistance. In fact, when onco-suppressor factors display low expression, chance of EMT-mediated chemoresistance increases. An interesting experiment has evaluated the function of SFRP5 in drug resistance in ovarian tumor; based on this experiment, epigenetic silencing of SFRP5 results in hyperactivation of Wnt to mediate EMT via TWIST upregulation and increases AKT2 levels in favor of drug resistance in ovarian tumor [150]. The interesting point is that even pathways related to EMT and Wnt can exhibit certain associations with the development of chemoresistance. Down-regulation of FILIP1L, an inhibitor of WNT, in ovarian tumor leads to SLUG upregulation and subsequent drug resistance development [151]. The interesting point is that when activation of Wnt occurs, it leads to 5-flourouracil resistance in colorectal tumor. The cadherin–catenin complex leads to simulation of β-catenin. However, Sanguisorba officinalis L. impairs nuclear translocation of β-catenin to diminish N-cadherin, vimentin and Snail levels, and enhance E-cadherin levels in increasing 5-flourouracil sensitivity in colorectal tumor [152]. Therefore, Wnt/EMT axis can modulate response of tumor cells to chemotherapy (Fig. 3).

## Pharmacological targeting of Wnt/β-catenin/EMT axis

Pharmacological inhibition of Wnt/EMT axis has offered new insight for cancer treatment to counter invasion and metastasis. Therefore, if molecular pathways related to Wnt/EMT are affected by these compounds, it can suppress invasion, as a factor mediating death in patients. O2-(2,4-dinitrophenyl) diazeniumdiolate is a new emerged compound in the treatment of HCC that its anti-tumor activity regarding Wnt regulation has been evaluated. O2-(2,4-dinitrophenyl) diazeniumdiolate suppresses Wnt/β-catenin axis to suppress EMT via reducing vimentin, Slug, Snail levels and increasing E-cadherin levels [153]. In order to increase potential in the modulation of Wnt/EMT axis, the combination of pharmacological compounds have been utilized in suppressing metastasis. Phytocannabinoid along with PARP1 inhibitor are capable of suppressing Wnt/β-catenin to minimize metastasis and mesenchymal phenotype of OC cells and EMT [154]. Cannabidiol (CBD) is well known in traditional Chinese medicine and there has been focus on its anti-tumor activity [155, 156]. One of the interesting points regarding CBD is its ability in the regulation of Wnt pathway. CBD promotes expression level of Axin1, suppresses Wnt/β-catenin pathway and reduces expression level of β-catenin target genes including APC and CK1 in EMT suppression in colorectal tumor [157].

In “Wnt/β-catenin signaling as a regulator of EMT in human cancers”, it was previously mentioned that increase in expression level of Wnt and induction of β-catenin can lead to EMT induction in HCC cells. Furthermore, related molecular pathways regulating Wnt/EMT axis in HCC were evaluated. Paris saponin H suppresses Wnt pathway in vitro and in vivo in HCC that changes expression level of EMT-related proteins such as vimentin and E-cadherin in favor of cancer invasion reduction [158]. However, one of the notes that should be considered during evaluation of experiments is that some of the studies have only focused on the regulation of EMT and Wnt separately in tumor cells and have not mentioned their association. For instance, wogonoside and Oldhamianoside II can suppress both Wnt/β-catenin and EMT in human cancers [159, 160], although future studies should focus on their association and modulation by these anti-tumor compounds.

**Fig. 4:**
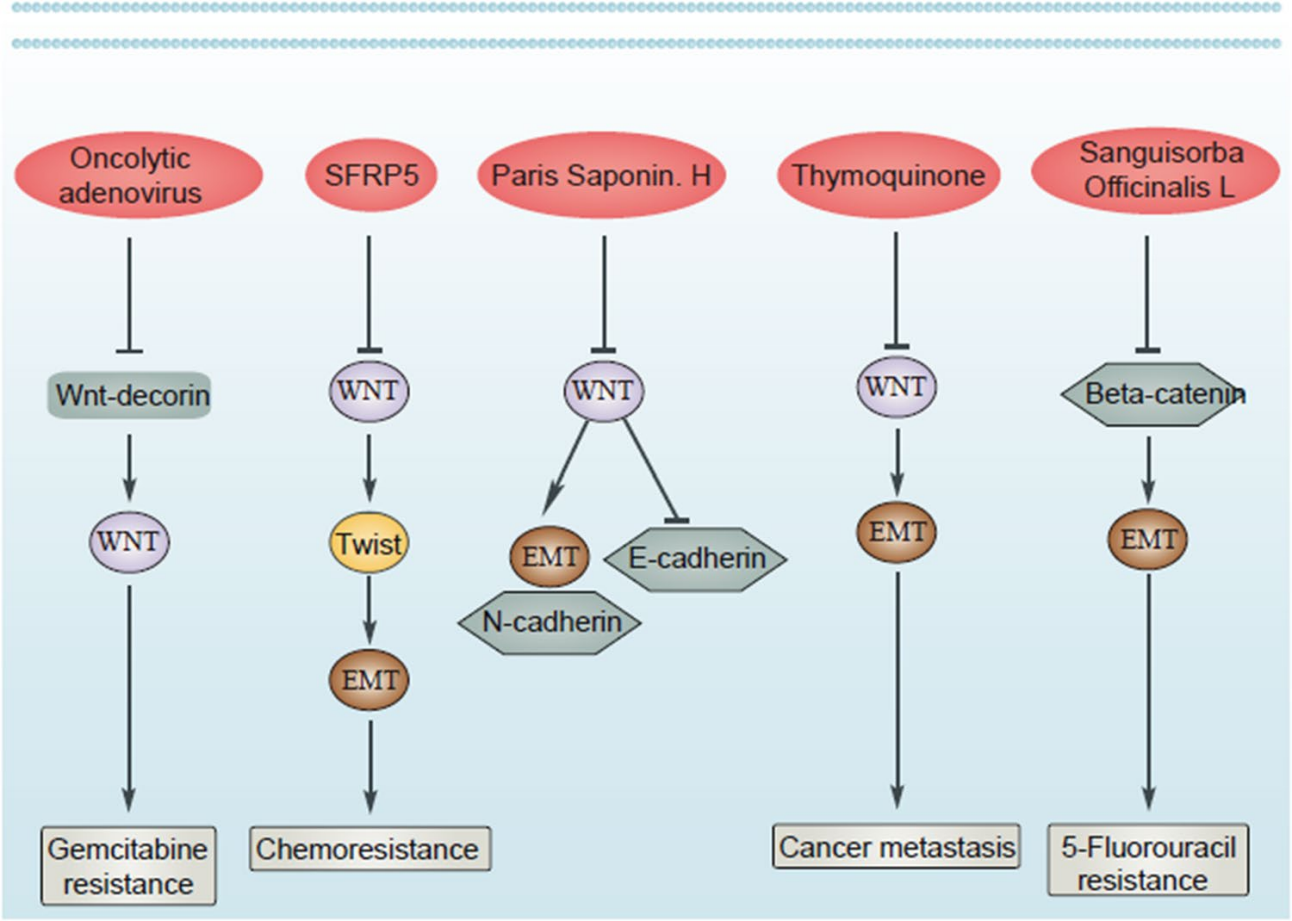
The role of Wnt/EMT in cancer drug resistance and its suppression by anti-cancer drugs. The application of oncolytic adenovirus allows to suppress the Wnt/EMT axis for reversing gemcitabine resistance. When Wnt expression increases, it stimulates Twist/EMT axis in mediating drug resistance. Moreover, suppression of Wnt by thymoquinone and other compounds can prevent cancer metastasis and chemoresistance

As mentioned, one of the main reasons of drug resistance is the induction of Wnt/EMT. In PCa, high expression of Wnt can result in malignant behavior and enzalutamide resistance. A combination of enzalutamide and 3,3ʹ-diindolylmethane can inhibit Wnt/β-catenin axis in EMT inhibition and suppressing EMT via enhancing E-cadherin levels and decreasing vimentin levels [161]. In fact, progression of PCa cells mainly depends on EMT and their enhanced metastasis relies on changes in phenotype and levels of mesenchymal and epithelial markers. 2'‑Hydroxyflavanone is capable of downregulating level of Wnt/β-catenin to suppress EMT in reducing progression of PCa cells [162]. Thymoquinone (TQ) is isolated from *Nigella sativa* and its pharmacological activities encompass antioxidant, anti-inflammation, immunomodulatory and anti-cancer [163–166]. Recently, TQ has been applied in the treatment of bladder cancer and by inhibiting Wnt/β-catenin/EMT axis, the metastasis of tumor cells significantly reduces [167]. Although bladder cancer is a multifactorial disease, tobacco smoke (TS), parasitic infection and radiation or chemical exposure have been considered as possible factors involved in its development. Accumulating evidence has shown an association between TS and development of bladder cancer [168]. Curcumin has been shown to suppress urocystic EMT and prevents acquisition of stemness in tumor cells. Curcumin suppresses Wnt/β-catenin axis to impair EMT, thus eliminating the development of TS-mediated bladder cancer [169]. Therefore, modulation and inhibition of Wnt/EMT axis by pharmacological compounds can greatly help in suppressing cancer invasion and metastasis [170–172] (Fig. 4).

## Nanoparticle-mediated regulation of Wnt/EMT axis in cancer metastasis: new visions

The introduction of nanostructures into the treatment of cancer revolutionized the ability to eradicate tumor cells. The pharmacological compounds suffer from poor pharmacokinetic profile and in spite of high potential in tumor suppression in vitro, their anti-cancer function reduces in vivo. Then, translation of such findings into clinic is a difficulty. Therefore, the targeted delivery of therapeutic for improving their accumulation at the tumor site has been suggested. Currently, the several conventional therapies have been introduced for cancer including chemotherapy and radiotherapy. Moreover, immunotherapy has been emerged for tumor suppression. However, the cancer cells stimulate the alternative molecular pathways to stimulate resistance into these therapies. The increasing evidences have shown that nanostructures can improve the ability of conventional therapies in cancer suppression, they reverse drug resistance and augment immunotherapy [173–176]. Since Wnt has been associated with EMT induction, the application of nanoparticles for the regulation of Wnt/EMT axis has been provided. The sweroside nanostructures have been introduced for the treatment of prostate cancer through increasing ROS generation and apoptosis. Moreover, these nanoparticles impair growth and invasion of tumor cells. They disrupt the stem cell features including CD33 and CD44. Moreover, sweroside nanoparticles impair TTCF/LEF activity to suppress β-catenin resulting down-regulation of c-Myc, cyclin D1, survivin and MMP-7. The down-regulation of Wnt/β-catenin by sweroside nanoparticles can also suppress the EMT-related markers in prostate cancer [177].

## Noncoding RNAs in regulation of Wnt/β-catenin

### microRNAs

microRNAs (miRNAs) are short and endogenous noncoding RNAs (ncRNAs) with length of 22–24 nucleotides. miRNAs are capable of binding to complementary components (3’-UTR) of mRNAs for degradation or translation suppression. This is known as post-transcriptional regulation of gene expression [178]. The dysregulation of miRNAs has been frequently observed in cancer and it can cause progression or suppression, based on function of miRNAs [179]. Since GSK-3β suppresses β-catenin, studies have focused on if there is any relationship between miRNAs and GSK-3β in EMT modulation in tumors. miR-1246 decreases GSK-3β levels by binding to its 3’-UTR in inducing β-catenin/EMT, resulting in enhanced cancer metastasis [180]. In KRAS-mutated colorectal tumor cells, expression level of miR-139-5p reduces and when poor expression of miR-139-5p occurs in cancer cells, their phenotype changes to an aggressive form. Interestingly, β-catenin may reduce miR-139-5p expression to induce EMT, resulting in elevated colorectal cancer invasion [181].

miR-27a is one of the factors that its exact function in cancer is not certain and miR-27a-3p sponging by circ-BCAR3 results in esophageal tumor progression [182], while down-regulation of miR-27a and miR-27b by circ-0000994 results in pancreatic tumor suppression [183], confirming dual function of miR-27a in cancers. miR-135 shows a contrast function compared to miR-27a and by decreasing SMAD3 expression, miR-135 suppresses TGF-β-mediated EMT in breast tumor [184]. Moreover, glycolysis and metabolic reprogramming in pancreatic tumor can be suppressed by miR-135 [185]. The EMT-TFs can be regulated by miRNAs in affecting progression of tumor cells. miR-519d inhibits Wnt/β-catenin and it reduces Twist1 expression to suppress EMT in gastric tumor [186]. However, the limitation of previous experiment is that it has not evaluated the correlation between Wnt and EMT regulated by miR-519d that can be focus of future studies.

In gastric tumor, high expression level of Wnt results in angiogenesis and metastasis, and silencing MED27 leads to inactivation of Wnt in retarding cancer progression [187]. FOXC1 upregulation results in induction of β-catenin in enhancing gastric tumor invasion [188]. Hence, metastasis of gastric tumor cells can be regulated by Wnt and a recent experiment has evaluated function of miR-330-3p in modulating invasion of gastric tumor. miR-330-3p is capable of impairing PRRX1-induced Wnt/β-catenin axis in reducing EMT and invasion of gastric tumor [189]. One of the interesting points is regulation of miRNAs by others factors in cancer cells. SOX17 and PAX8 physically cooperate in increasing ovarian tumor progression that it can be suppressed by small molecule inhibitors [190]. Moreover, reduced SOX17 expression by miR-200a-3p can result in increase in proliferation and invasion of tumor cells [191].

One important feature of miRNAs is their enrichment in exosomes, as small extracellular vesicles with 40–120 nm in diameter and secreted from many cells into body fluids [192–195]. When exosomes were discovered, it was believed that these structures contribute to removal of intracellular wastes [196], while recent studies have shown that exosomes are potential regulators of carcinogenesis [197]. Low expression level of miR-7-5p in exosomes derived from breast tumor cells can result in enhanced metastasis. However, if exosomes derived from breast tumor cells have high levels of miR-7-5p, it can lead to the regulation of atypical WNT in which reducing RYK expression to favor JNK phosphorylation, resulting in c-Jun protein enhancement and subsequent EMT inhibition in decreasing cancer metastasis [198]. The catch point is that exosomal miRNAs can regulate TME components such as cancer-associated fibroblasts (CAFs). miR-146a demonstrates enrichment in exosomes derived from breast tumor cells. Through reduction in TXNIP levels, miR-146a induces Wnt/β-catenin axis in the promotion of metastasis and EMT-related protein expression such as vimentin and N-cadherin [199].

### Long noncoding RNAs

Long non-coding RNAs (lncRNAs) are transcribed by RNA polymerase II transcripts with length more than 200 nucleotides [200]. In addition to implication in the modulation of biological processes, their function in cancer is also of importance [201, 202]. LncRNAs can exert sponging effect in reducing miRNA expression and modulating biological events. Furthermore, various molecular pathways are affected by lncRNAs present in cytoplasm and nucleus, and function of lncRNAs is different based on the location that those in nucleus provide chromatin remodeling and cytoplasmic lncRNAs mediate miRNA interaction. Wnt regulation by lncRNAs can affect metastasis of tumors. miR-106a-3p suppresses APC expression in gastric tumor, while LINC01133 sponges miR-106a-3p to increase APC expression. Then, APC suppresses β-catenin in reducing invasion and EMT [203]. LINC01089 suppresses EMT and invasion in lung tumor and for this purpose, it follows a complicated pathway in which LINC01089 sponges miR-27a to increase SFRP1. Then, suppression of Wnt/β-catenin axis is observed to inhibit EMT in reducing cancer invasion and metastasis [204]. In contrast, lncRNAs can promote invasion of tumor cells through induction of EMT mechanism. For instance, lncRNA NORAD decreases miR-30a-5p expression as a way to promote RAB11A levels in the induction of Wnt/β-catenin, resulting in EMT and enhanced cancer invasion [205]. Besides, lncRNA HOXD-AS1 sponges miR-133a-3p to induce Wnt/β-catenin for EMT induction and promoting metastasis of ovarian tumor [206]. Therefore, lncRNAs are important regulators of Wnt/EMT axis in human cancers.

### Circular RNAs

**Fig. 5:**
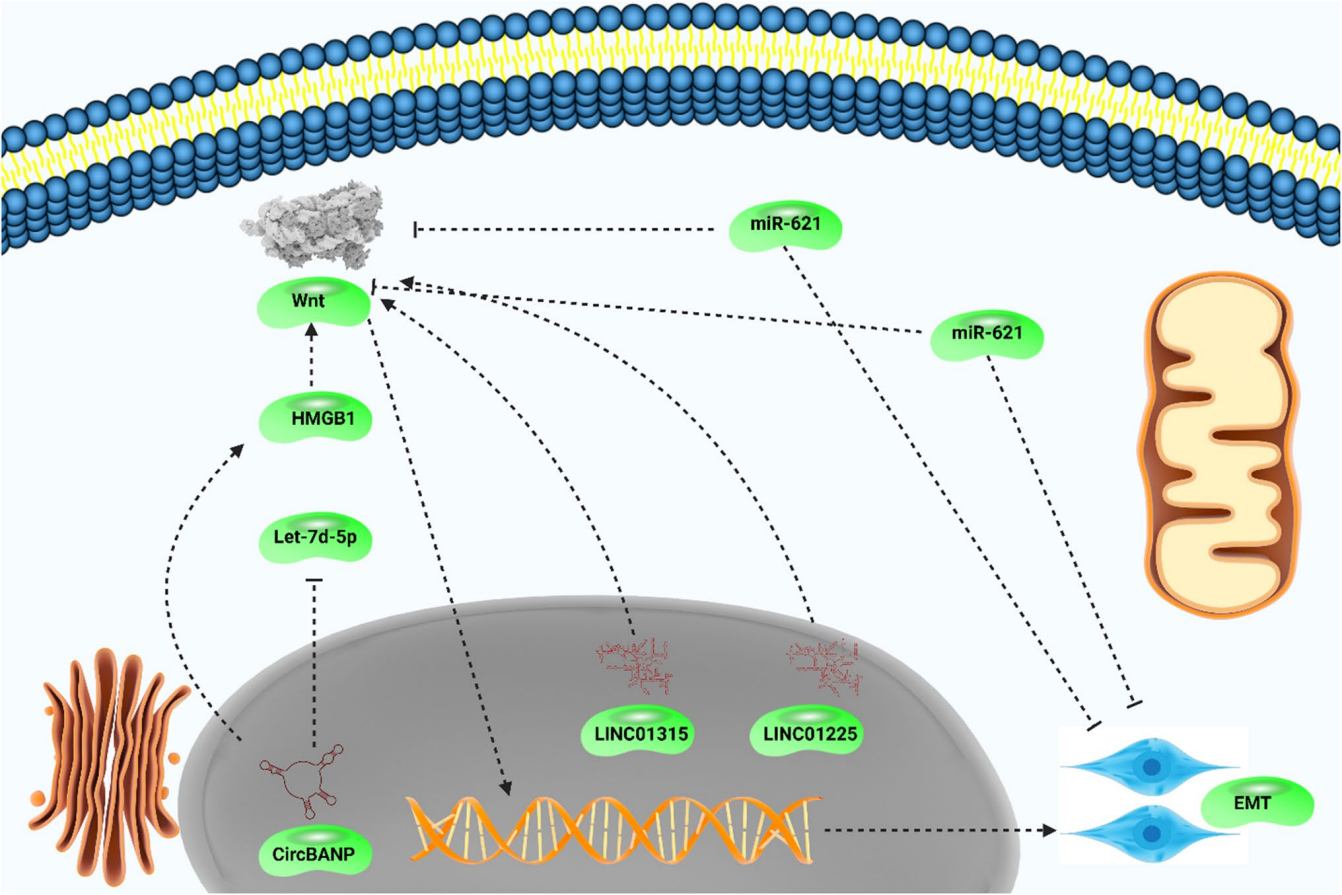
The non-coding RNAs regulating Wnt/EMT axis in human cancers. The functional miRNAs present in the cytoplasm, while lncRNAs and circRNAs can be in cytoplasm or nucleus. Moreover, the inhibition of EMT by non-coding RNAs suppresses metastasis and EMT

Circular RNAs (circRNAs) are other factors that are considered as special ncRNAs and they are ubiquitous in human cells [207]. CircRNAs are new endogenous ncRNAs that were adjusted to be pivotal regulators of various biological mechanisms [208] and their unique roles in tumors have been interesting. Increasing evidence reveals that circRNAs may modulate EMT in cancers [209]. More importantly, circRNAs can decrease miRNA expression via sponging [210]. The overall aim of current section is to evaluate role of circRNAs in EMT regulation via targeting Wnt. Circ-0007059 shows function in suppression of lung tumor growth and metastasis, and its ability in decreasing metastasis is due to EMT suppression. The hsa-circ-0007059 decreases miR-378 expression to inhibit Wnt/β-catenin and ERK1/2 in reducing vimentin, Twist, and ZEB1 levels to suppress EMT [211]. On the other hand, high expression level of circ-000984 results in enhanced invasion of lung tumor that is related to increasing β-catenin levels in mediating EMT [212]. SP1 is a transcription factor that modulates intracellular gene expression, and its dysregulation has resulted in increased carcinogenesis, especially colorectal cancer [213]. Since colorectal tumor is one of the leading causes of death, ample efforts have been engaged towards understanding circRNAs in the regulation of Wnt and EMT in this malignant tumor. Hsa-circ-0001666 can diminish malignancy of colorectal tumor and mechanistically, circ-0001666 decreases miR-576-5p expression to upregulate PCDH10, to induce Wnt pathway and to increase EMT [214]. Circ-0067934 is one of the new emerging factors in cancer that its upregulation prevents ferroptosis in thyroid tumor [215] and by decreasing JNK phosphorylation, it can result in cisplatin resistance [216]. More than one molecular pathway can be simultaneously regulated by circ-0067934 and notably, this circRNA induces Wnt/β-catenin pathway and promotes KLF8 expression via miR-1182 down-regulation to induce EMT and metastasis in lung tumor [217]. Although each study provides a new insight and pathway in which Wnt/EMT axis is regulated by circRNAs, many of them share a similar function and that is miRNA sponging by circRNAs. Circ-0082182 promotes progression and metastasis in colorectal tumor that is mediated by miR-411 and miR-1205 suppression via sponging to stimulate Wnt/β-catenin pathway in EMT induction [218]. Table 4 and Fig. 5 summarize the role of ncRNAs in Wnt/EMT axis regulation in human cancers.

**Table 4:** The modulation of Wnt/EMT axis by ncRNAs in human cancers

Non-coding RNA	Pathway	Action mechanism	Refs.
miR-621/Wnt/EMT	Colorectal cancer	miR-621 suppresses EMT via Wnt down-regulation	[219]
LINC01315/Wnt/EMT	Colorectal cancer	LINC01315 induces EMT via Wnt upregulation	[220]
CircZFR/miR-3619-5p/Wnt	Hepatocellular carcinoma	CircZFR sponges miR-3619-5p and induces Wnt pathway to promote invasion via EMT induction	[221]
miR-194/Wnt/EMT	Hepatocellular carcinoma	miR-194 suppresses Wnt pathway to reduce progression of tumor cells and to inhibit EMT	[222]
CARLo-7/Wnt/EMT	Bladder cancer	CARLo-7 induces Wnt/EMT axis in increasing metastasis	[223]
DLX6-AS1/Wnt/EMT	Bladder cancer	DLX6-AS1 simulates Wnt/EMT axis	[224]
LSINCT5/NCYM/Wnt/EMT	Bladder cancer	LSINCT5 interacts with NCYM in increasing Wnt expression to mediate EMT	[225]
PlncRNA-1/Wnt/EMT	Colorectal cancer	PlncRNA-1 activates Wnt in increasing cancer invasion and EMT induction	[226]
ADAMTS9-AS1/Wnt	Colorectal cancer	ADAMTS9-AS1 suppresses Wnt/EMT axis in reducing invasion and metastasis of tumor cells	[227]
LncRNA-SRA/Wnt/EMT	Endometrial cancer	SRA induces Wnt/EMT axis in increasing cancer progression	[228]
LINC01225/Wnt/EMT	Gastric cancer	LINC012225 induces Wnt signaling in increasing cancer progression through EMT induction	[229]
Circ_0003789/Wnt/EMT	Gastric cancer	Circ_0003789 stimulates Wnt/EMT axis	[230]
JPX/miR-33a-5p/Twist1	Lung cancer	JPX sponges miR-33a-5p to increase Twist1 expression Twist1 induces Wnt and promotes EMT	[231]
miR-516a-3p/Pygo2/Wnt/EMT	Breast cancer	miR-516a-3p suppresses Pygo2/Wnt axis in EMT inhibition	[232]
miR-15a-3p/Wnt/EMT	Prostate cancer	miR-15a-3p suppresses Wnt/EMT axis	[233]
LncRNA-MIR17HG/miR-17/miR-18a/Wnt/EMT	Colon cancer	LncRNA-MIR17HG promoted miR-17 and miR-18a expression to induce Wnt/EMT	[234]
miR-370-3p/Wnt7a/EMT	Bladder cancer	miR-370-3p suppresses Wnt7a expression to suppress EMT	[235]

## Conclusion and remarks

The regulation of molecular pathways in cancer represents one of the important aspects of research for biologists to track down the interplay among these signaling cascades in the regulation of carcinogenesis. A given form of cancer can display distinct features and characteristics such as growth, invasion and chemoresistance. Each hallmark of cancer may intertwin with one another. For instance, increase in proliferation and invasion of tumor cells promotes their malignancy, resulting in the development of therapeutic resistance. However, among the different hallmarks, increase in metastasis is an important aspect that causes high death among patients and can mediate therapy failure. The process of metastasis is complicated and one important mechanism facilitating tumor invasion is EMT mechanism. Wnt/β-catenin is the most well-known one among various regulators of EMT. Due to tumor-promoting property of Wnt, it can induce EMT in increasing tumor progression. High expression level of Wnt occurs during progression of tumor cells and this is in favor of cancer invasion. Wnt/β-catenin can mediate EMT in various cancers including brain, hematological, urological, gynecological and gastrointestinal tumors. Therefore, an optimal strategy is to directly suppress Wnt pathway or inhibit EMT as another key player in the progression of tumor. Since both Wnt and EMT mechanisms result in the drug resistance in human cancers, studies demonstrated that EMT induction by Wnt can also lead to chemoresistance. Therefore, inhibition of Wnt/EMT axis would be the important next step to overcome drug resistance in cancers. The modulation of Wnt/EMT axis by non-coding RNAs is a hot topic recent year and since Wnt has bindings site for non-coding RNAs, Wnt/EMT axis can be modulated by these RNA transcripts. Furthermore, pharmacological compounds and nanostructures are capable of Wnt/EMT suppression in reducing invasion and metastasis of tumor cells. 

## Data Availability

Not applicable.
