# Editorial Expression of Concern: Wnt/β-catenin-driven EMT regulation in human cancers

**DOI:** 10.1007/s00018-026-06193-2

**Published:** 2026-04-10

**Authors:** Lin Yang, Chengxin Chen, Milad Ashrafizadeh, Yu Tian, Ranran Sun, Wenhua Xue

**Affiliations:** 1 Department of Pharmacy, The First Affiliated Hospital of Zhengzhou University, Zhengzhou, 450052 Henan People’s Republic of China; 2 Department of Hepatobiliary Surgery, Xianyang Central Hospital, Xianyang, 712000 Shaanxi China; 3 Zhongshan Hospital, Shanghai Institute of Cardiovascular Diseases, Fudan University, Shanghai, 200032 China; 4 School of Public Health, Benedictine University, Lisle, USA; 5 Precision Medicine Center, The First Affiliated Hospital of Zhengzhou University, Zhengzhou, Henan China

**Editorial Expression of Concern: Cellular and Molecular Life Sciences (2024) 81:79**

10.1007/s00018-023-05099-7

The Editors-in-Chief are issuing an Expression of Concern. 

Post-publication review found issues such as unreliable reporting of the primary articles cited and information that is incomplete. These include Inaccurate depiction of WNT pathway regulation (Fig. 1) and several citations, including references 19 and 20, that do not directly support statements made in the paper. The readers are asked to interpret the findings with caution.

Milad Ashrafizadeh agrees to this Expression of Concern. Wenhua Xue, Lin Yang, Chengxin Chen, Yu Tian and Ranran Sun did not respond to correspondence from the Editor about this Expression of Concern.

